# Authors’ response: COVID-19: how accurate are seroprevalence studies?

**DOI:** 10.2807/1560-7917.ES.2020.25.30.2001437

**Published:** 2020-07-30

**Authors:** Irene Cassaniti, Elena Percivalle, Antonella Sarasini, Giuseppe Cambiè, Gherard Batisti Biffignandi, Danilo Cereda, Fausto Baldanti

**Affiliations:** 1 Molecular Virology Unit, Microbiology and Virology Department, IRCCS Policlinico San Matteo, Pavia, Italy; 2 Department of Clinical Surgical Diagnostic and Pediatric Sciences, University of Pavia, Pavia, Italy; 3 Immunohematology and Transfusion Medicine Unit, Ospedale Maggiore di Lodi, Lodi, Italy; 4 Lombardy Region, Directorate General for Health, UO Prevenzione, Milan, Italy

**Keywords:** Italy, viral infections, respiratory infections, respiratory viruses, surveillance, coronavirus disease, COVID-19, SARS-CoV-2, seroprevalence

**To the editor**: We are grateful to Kamran Kadkhoda for the comments provided in his letter [1]. With this response, we wish to clarify the concerns raised and provide some more insights.

The estimated 4.7% seroprevalence to severe acute respiratory syndrome coronavirus 2 (SARS-CoV-2) is based upon an assumption (only 20% of cases are tested for RNA). Lombardy has ca 10 million inhabitants and from 20 February to 22 July 2020, 1,217,819 nasal swabs were performed (for ca 12% of the total population). Of these, 88,824 were positive (7.2%) [2].

Lombardy has not been homogenously affected by COVID-19: two major outbreaks were recorded in Lodi-Cremona and Bergamo-Brescia, while other areas of Lombardy (Varese, for instance) were little affected. From 20 February to 30 March, 3,387 nasal swabs were performed in the Lodi Red Zone and 991 (29.2%) were positive. These data strongly support our previous findings [3].

In fact, our paper [3] did not deal with the prevalence of neutralising antibodies in the whole of Lombardy, but with the prevalence in one of the two ‘Red Zones’ which represent the epicentre of one of the two major coronavirus disease (COVID-19) outbreaks in Lombardy. Indeed, we were not surprised that COVID-19 prevalence was higher than in other parts of Lombardy or with respect to the mean regional value.

We have recently completed a further analysis on 1,139 additional blood donors from the Lodi Red Zone and the adjacent Lodi metropolitan and suburban area using in parallel a commercial assay detecting S1/S2 IgG [4] and this neutralisation (NT) assay. The data essentially confirm and extend our previous observation since as many as 22.2% blood donors form this larger area showed a positive (S1/S2 IgG) response and 21.6% had a positive NT response (data not shown).

As for the specificity and sensitivity of our NT assay, they were determined in advance using standard procedures [3]. The assay was also challenged against sera from 10 individuals with known positivity for human coronavirus, without showing cross-reactivity [3].

Finally, as pointed out by Kamran Kadkhoda, a degree of cross reactivity between components (Zika and dengue viruses) of other virus families (flaviviruses) has been reported previously [5].

In order to evaluate the sequence similarity of SARS-CoV-2 with human alpha- and beta-coronaviruses in comparison with components of the flavivirus family, we downloaded 12 representative genomes of these two groups and performed an average nucleotide identity calculation with Hadamard weight for coverage on 250 nt fragments using the PyAni tool (https://github.com/widdowquinn/pyani). We plotted the results using pheatmap and the resulting heatmap clearly shows that SARS-CoV-2 presents a very low Hadamard correlation with other human coronaviruses.

The genetic similarity among flaviviruses (median value: 0.10449) is significantly higher than among coronaviruses (median value: 0.03168; p: 0.000494) (Figure). In particular, SARS-CoV-2 was less genetically similar to human beta-coronaviruses (OC43 and HKU1) than Zika virus to dengue 1–4 viruses. The genetic similarity of SARS-Cov-2 to human alpha-coronavirus (NL63 and 229E) is even less pronounced (Figure).

**Figure:**
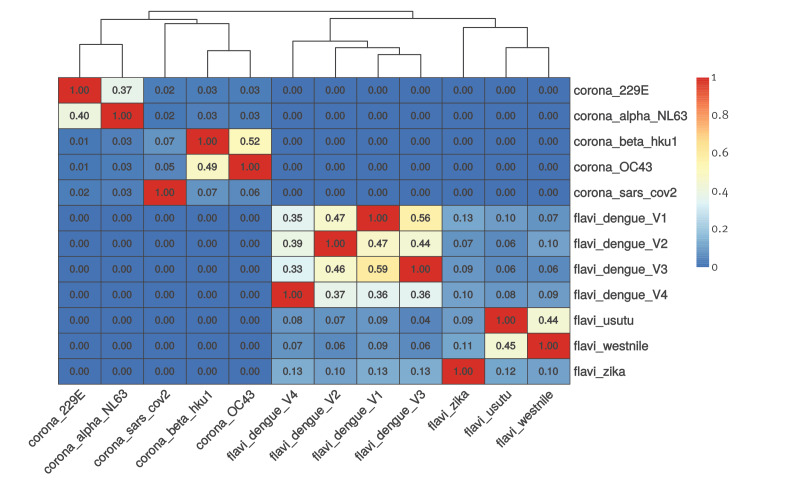
Heatmap of the Hadamard distances within the complete genomes of flaviviruses and coronaviruses
